# Logistic Regression Ensemble Classifier for Intrusion Detection System in Internet of Things

**DOI:** 10.3390/s23239583

**Published:** 2023-12-03

**Authors:** Silpa Chalichalamala, Niranjana Govindan, Ramani Kasarapu

**Affiliations:** 1 Department of Computer Science and Engineering, SRM Institute of Science and Technology, Kattankulathur, Chennai 603203, India; silpa.c8@gmail.com; 2 Department of Computing Technologies, School of Computing, SRM Institute of Science and Technology, Kattankulathur, Chennai 603203, India; 3 School of Computing, Mohan Babu University (Erstwhile Sree Vidyanikethan Engineering College), Tirupati 517102, India; hod_it@vidyanikethan.edu

**Keywords:** adaptive synthetic sampling, Internet of Things, intrusion detection system, logistic regression-based ensemble classifier, recursive feature elimination

## Abstract

The Internet of Things (IoT) is a powerful technology that connect its users worldwide with everyday objects without any human interference. On the contrary, the utilization of IoT infrastructure in different fields such as smart homes, healthcare and transportation also raises potential risks of attacks and anomalies caused through node security breaches. Therefore, an Intrusion Detection System (IDS) must be developed to largely scale up the security of IoT technologies. This paper proposes a Logistic Regression based Ensemble Classifier (LREC) for effective IDS implementation. The LREC combines AdaBoost and Random Forest (RF) to develop an effective classifier using the iterative ensemble approach. The issue of data imbalance is avoided by using the adaptive synthetic sampling (ADASYN) approach. Further, inappropriate features are eliminated using recursive feature elimination (RFE). There are two different datasets, namely BoT-IoT and TON-IoT, for analyzing the proposed RFE-LREC method. The RFE-LREC is analyzed on the basis of accuracy, recall, precision, F1-score, false alarm rate (FAR), receiver operating characteristic (ROC) curve, true negative rate (TNR) and Matthews correlation coefficient (MCC). The existing researches, namely NetFlow-based feature set, TL-IDS and LSTM, are used to compare with the RFE-LREC. The classification accuracy of RFE-LREC for the BoT-IoT dataset is 99.99%, which is higher when compared to those of TL-IDS and LSTM.

## 1. Introduction

The Internet of Things (IoT) is generally a smart network that enables seamless internet connections across a wide array of physical devices/components/entities that comprise the IoT smart network. It does so with the goal of broadcasting data from anywhere in the world. Accordingly, any user has the capacity to access any kind of information pertaining to their requirements without any constraints of time and location. The entities or objects that are involved within an IoT network system are wirelessly linked using smart tiny sensors. Therefore, IoT devices have the capacity to interact with others without any human intervention [1]. IoT devices perform across numerous environments to achieve various objectives, leading to the development of several computing and communication technologies used in healthcare, military, agriculture, business and education [2]. A key component in providing security to IoT networks is the detection of intrusions [3]. An Intrusion Detection System (IDS) is an approach developed to ensure network security by detecting, preventing and eliminating unauthorized access during communication. IDS plays an essential role in making the network secure and safe, as its main objective is to guarantee the privacy, accessibility and authenticity of the system [4]. An IDS actively monitors a network for any malicious activity and alerts the system administrator if found. IoT devices are small and portable, which makes them perfect for remote regions [5]. Some of the applications of the IoT, including retail environments, intelligent buildings, smart cities and interconnected vehicles, are susceptible to malicious attacks. Therefore, it is necessary to implement security enhancements such as secure booting, device authentication and access control [6]. The IoT has become an attractive sector for cyberattacks due to its business growth and financial potential. This is the primary reason for the rapid increase in cyberattacks against IoT devices [7]. Proactive network security defenses are required to protect essential assets and data because IoT attack vectors have the potential to result in successful security breaches [8].

The IDS continuously monitors incoming and outgoing network traffic produced by IoT devices to search for any signs of cyberattacks. The two types of attack detection techniques are anomaly based and signature based [9]. The communication link between IoT devices is susceptible to a number of security risks, including data integrity attacks, Distributed Denial of Service (DDoS), session hijacking and man-in-the-middle attacks [10]. Continuous developments in devices, applications and services of IoT have led to increased vulnerabilities in IoT systems [11]. Hence, with the lack of fundamental security processes, the IoT devices become vulnerable targets for attackers and hackers. For example, the IoT is attacked and hacked by botnets that are utilized for initiating DDoS attacks [12]. The enormous internet deployments of IoT devices are associated with an increase in cyberattacks [13]. The maintenance of a minimal processing load in IoT devices is one of the main goals of the technology. Consequently, Host Intrusion Detection Systems (HIDS) are frequently avoided in the IoT ecosystem due to resource-intensive activities such as file or process monitoring [14]. There are numerous efforts made to increase IoT security, including the adoption of complex access control mechanisms for data confidentiality, the application of encryption on data transported in networks and various privacy and trust rules among users and IoT devices [15].

The contributions of this study are typified as follows:

- An LREC classifier that includes the combination of AdaBoost and RF is used for performing effective classification of intrusions. The integration of AdaBoost and RF, according to the iterative ensemble approach, builds an effective classifier.
- The issue of data imbalance in the input data is avoided using the ADASYN. The ADASYN is specifically chosen because it effectively controls the network traffic with severe data imbalance. Further, the RFE is utilized for removing the feature with less information so that the prediction is enhanced.

The article is organized as follows: The related works about the IDS are given in Section 2. A detailed explanation of RFE-LREC is given in Section 3, whereas the simulation results are presented in Section 4. Further, this research is concluded in Section 5.

## 2. Related Work

Sarhan et al. [16] implemented a NetFlow-based standard feature set for the Network Intrusion Detection System (NIDS) datasets of CSE-CICIDS2018, BoT-IoT, UNSW-NB15 and ToN-IoT. Their publicly accessible packet capture (pcap) data and ground truth events were used to extract features and tag methods. Due to the widespread availability of effective collection and NetFlow exporters, the implemented NetFlow-based feature sets had the benefit of being scalable and highly flexible. However, NetFlow was unable to carry out such an exhaustive and rigorous evaluation due to the restricted deployment of ML-based NIDSs in real-world network settings.

Disha and Waheed [17] presented the Gini Impurity-based Weighted Random Forest (GIWRF) to choose significant and pertinent features based on the importance score. The Gini Impurity criteria were used to split the trees, and the weight in the Random Forest algorithm was adjusted for unbalanced class distribution to produce the feature importance score. The presented method detected the intrusions effectively in the imbalanced data class from UNSW-NB15 and ToN-IoT datasets. The developed GIWRF was only suitable for binary classes as it failed to perform the multiclass classification.

Rodríguez et al. [18] implemented an efficient intrusion detection method with UNSW-NB15 dataset based on transfer learning (TL), model refinement and knowledge transfer to identify a wide range of zero-day attacks with imbalanced and scarce datasets. A test dataset with five kinds of innovative attacks was created to assess the TL-based ID framework. With decreased representation in the dataset, the TL-based IDS showed greatly increased efficacy in the detection of zero-day and known threats. Nevertheless, transfer learning, model refinement and knowledge transfer were computationally intensive processes, requiring significant amounts of resources such as processing power and training data.

Zhang et al. [19] implemented a novel network anomaly detection algorithm in terms of multiclass balancing and semi-supervised learning to efficiently detect numerous kinds of anomalous traffic data in actual network environments. This anomaly detection was performed using UNSW-NB15, NSL-KDD and ToNIoT datasets. To develop the principle of consistent distribution among unlabeled and labeled data, the multiclass split balancing and the adaptive confidence threshold function were employed. The implemented method improved the detection performances by using the collaborative rotation forest algorithm to learn from unlabeled and labeled samples. Yet, with remarkable performance and broad applicability, the novel network anomaly detection algorithm was inapplicable to datasets with erratic distribution.

Asgharzadeh et al. [20] presented an IoT Feature Extraction Convolutional Neural Network (IoTFECNN) with hybrid layers to extract both low-level and high-level characteristics and identify IoT anomalies from TON-IoT and NSL-KDD datasets. For effective feature selection, the Binary Multi-objective Enhanced Capuchin Search Algorithm (BMECapSA) was developed. A new hybrid technique, CNN-BMECapSA-RF, was developed by incorporating the IoTFECNN and BMECapSA methods to improve the IoT’s ability to detect anomalies with greater accuracy and precision. Due to improved feature extraction, selection and robust classification, the CNN-BMECapSA-RF technique greatly identified abnormalities in the IoT across all criteria. Anyhow, the execution time and complexity of connecting the BMECapSE to a classifier during the execution created difficulties.

Banaamah and Ahmad [21] implemented deep learning methods based on Long Short-Term Memory (LSTM), Conventional Neural Networks (CNN) and Gated Recurrent Units (GRU) to detect intrusions using Bot-IoT dataset. The CNN, LSTM and GRU methods were evaluated using a BoT-IoT standard dataset for IoT intrusion detection. The implemented method effectively performed the intrusion detection. Here, the LSTM provided better performance than both CNN and GRU because, for training and inference, CNN utilized complex computations that consumed a lot of resources and required more powerful hardware. 

Lazzarini et al. [22] implemented a Deep Integrated Stacking for the IoT (DIS-IoT) for intrusion detection, using the TON-IoT dataset based on a stacking ensemble of deep learning (DL) models. DIS-IoT integrated four various DL models—: Deep Neural Network (DNN), MultiLayer Perceptron (MLP), Convolutional Neural Network (CNN) and Long Short-Term Memory (LSTM)—to make use of the numerous classification properties. The DIS-IoT performed effectively in both binary and multiclass classification for intrusion detection. However, DIS-IoT was an ensemble of four different methods requiring a large number of resources to run directly on low-power IoT devices.

Fatani et al. [23] presented novel feature extraction and selection techniques for creating an IDS system utilizing the benefits of a Swarm Intelligence (SI) algorithm, with KDD99, CIC2017, BoT-IoT and NSL-KDD datasets. For the purpose of extracting relevant features from the datasets, a feature extraction method based on the Conventional Neural Network (CNN) was created. Then, utilizing the recently developed SI algorithm, the Aquila Optimizer (AQU) provided an alternative feature selection technique to choose optimal features and improve the accuracy of classification. In both binary and multiclass classification situations, AQU had a great ability to boost the intrusion detection efficiency, but it still required a lot of computing power, particularly when applied to large datasets or complicated feature spaces.

Zeeshan et al. [24] introduced a Protocol-Based Deep Intrusion Detection (PB-DID) architecture for Denial of Service (DoS) and Distributed DOS attacks by analyzing the features of two recent benchmark datasets, BoT-IoT and UNSW-NB15. The typical features of the low and Transmission Control Protocol (TCP) categories in both datasets were examined and merged in PB-DID. The PB-DID significantly minimized the processing time by using minimal features for training and classification. Yet, to make both datasets compatible for testing, it was required to calculate a few UNSW-NB15 features that were absent in the BoT-IoT and vice versa.

Fatani et al. [25] introduced an efficient AI-based mechanism for the system of intrusion detection in IoT, utilizing the benefits of a deep learning and MetaHeuristics (MH) optimization algorithm. There were four different datasets, NSL-KDD, KDDCup-99, CICIDS-2017 and BoT-IoT, that were considered in this work. To extract relevant features, a feature extraction method based on the Conventional Neural Network (CNN) was created. To increase the balance between the exploration and exploitation phases, the new feature selection method employed a new form of the Transient Search Optimization (TSO) algorithm using the differential evolution (DE) algorithm. The TSODE-selected features amplified the classifier’s efficiency in detecting each of the attack classes. Despite that, the complexity of the algorithm increased when the DE algorithm was combined with TSO.

Halim et al. [26] presented Genetic Algorithm (GA)-based feature selection for avoiding the dimensionality curse in the datasets Bot-IoT, CIRA-CIC-DOHBrw-2020 and UNSW-NB15. The developed GA was used to choose adequate features from the data for improvement in the accuracy. But the developed GA required a larger amount of reproduction operations and repetitive iterations.

Zhou et al. [27] developed the IDS according to the incremental Long Short-Term Memory (LSTM) using CICIDS2017 and UNSW-NB15 datasets. The increment, i.e., product of function and derivative of LSTM, was used for obtaining the dynamic information of traffic. Next, the state variation was used in LSTM to consider that as incremental LSTM. The developed incremental LSTM was used to choose the optimal feature subset for further accuracy enhancement.

## 3. RFE-LREC Method

**Figure 1:**
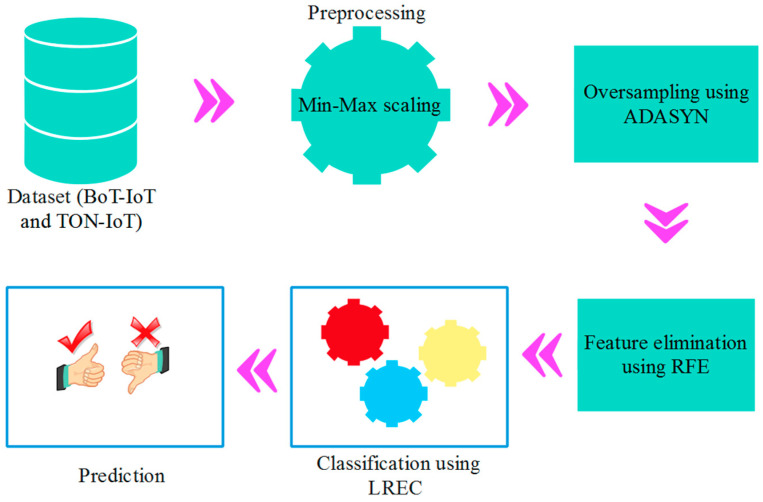
Block diagram of RFE-LREC.

This research study proposes the development of a robust Intrusion Detection System using an efficient ensemble classification. The important processes involved in this research are (1) dataset acquisition, (2) preprocessing, (3) oversampling using ADASYN, (4) feature elimination using RFE and (5) ensemble classification. The issue of data imbalance is solved by using the ADASYN, while the irrelevant features from the overall feature set are excluded by using the RFE. Further, the logistic regression-based ensemble classifier (LREC) is used to increase the prediction performance of network intrusion. Figure 1 shows the block diagram of RFE-LREC.

### 3.1. Dataset Acquisition

The analyses in this research are carried out using two different datasets, which are BoT-IoT [28] and TON-IoT [29].

- The BoT-IoT dataset is generated in the IoT lab of the University of New South Wales (UNSW) based on a realistic network environment. The BoT-IoT dataset is suitably considered when concentration is on the discovery of IoT devices that are compromised or are acting in a malicious or anomalous way, often referred to as “bots.” The BoT-IoT dataset comprises network traffic information that obtains the different behaviors of IoT devices, such as sensors, smart cameras and remaining linked devices, in a controlled and simulated environment. This data comprises both normal and malicious behaviors, making it a valuable resource to train and estimate an IDS. It has 72 million records of cyberattacks comprising Denial of Service (DoS), Distributed DoS (DDoS), ransomware and reconnaissance. The raw information is available in the pcap field format with a size of 16.7 Gigabits. Further, the UNSW provides the BoT-IoT in two formats, argus and CSV. In the argus format, packets are gathered into flows according to feature vector, while the packet features and its respective classes are provided in the CSV format.
- The TON-IoT dataset was introduced by the makers of the BoT-IoT dataset to provide a comprehensive dataset that comprises normal and various attack types that threaten the industrial IoT (IIOT). The TON-IoT is created from certain current technologies such as multiple clouding layers, fog and edge. It has 22,339,021 network instances in CSV, pcap and Argus formats.

### 3.2. Preprocessing Using Min–Max Scaling

The data obtained from the datasets is preprocessed under min–max scaling to convert and rescale the values between the range of 0 and 1 using Equation (1).

(1) $$X^{\prime }=\frac{X-X_{min}}{X_{max}-X_{min}}$$

where X is the input, $X^{\prime }$ is preprocessed output and $X_{min}$ and $X_{max}$ are the minimum and maximum values of input.

### 3.3. Oversampling Using ADASYN

Adaptive synthetic sampling (ADASYN) is an adaptive oversampling approach that depends on the minority class samples. This ADASYN generates a huge amount of samples when there is less density and generates fewer samples when there is huge density. This characteristic has the benefit of adaptively shifting the decision boundaries when it is difficult to observe the samples. Hence, the ADASYN is effective in handling network traffic even with the issue of severe data imbalance.

The steps processed in ADASYN are as follows:

1. The G amount of samples that are required to be synthesized is computed as denoted in Equation (2).
  (2) $$G=\left(n_{b}-n_{s}\right)\times \beta$$
  where the minority and majority samples are denoted as $n_{s}$ and $n_{b}$, respectively, and $\beta \in \left(0,1\right)$.
2. K amount of neighbors is computed by Euclidean distance denoted by $r_{i}$, i.e., the ratio of majority class samples existing in the neighborhood for each minority sample. Equation (3) expresses the $r_{i}$.
  (3) $$r_{i}=k/K$$
  where k is the majority class sample in the current neighbor and K denotes the current amount of neighbors.
3. The g amount of samples required to be synthesized for each minority sample is computed in Equation (4), and then the samples are synthesized based on Equation (5).
  (4) $$g=G\times r_{i}$$
  (5) $$Z_{i}=X_{i}+\left(X_{Zi}-X_{i}\right)\times \lambda$$
  where the amount of samples needed to be synthesized is denoted as g; a new synthesized sample is denoted as $Z_{i}$; the current minority sample is denoted as $X_{i}$; a random minority sample between k neighbors is $X_{Zi}$; and $\lambda \in \left(0,1\right)$.

### 3.4. Feature Elimination Using RFE

The synthesized sample $\left(Z\right)$ from ADASYN is given as input to the RFE, wherein the feature selection is performed through sequential backward elimination. The RFE starts with an overall group of features and removes one feature at a time. The feature with less information is eliminated each time. The usage of binary SVM in RFE is for effectively ranking the feature by discovering the optimal hyperplane that divides the classes in the feature space. The process of optimal hyperplane search considers the most discriminative features, which helps to capture highly relevant features. RFE, when applied to binary SVM, offers numerous advantages. It systematically computes feature rankings, providing insights into their significance. By progressively eliminating the least influential features, RFE streamlines the dataset, reducing the dimensionality. This not only enhances model performance, but it also streamlines and accelerates the computation. In the scenario of this research study, the top 10 best features are used, which achieves an efficient balance between information retention and model simplification. The result is a more interpretable and robust model that well generalizes any new data. RFE, in conjunction with SVM, proficiently selects the most relevant features, contributing to improved predictive accuracy along with a streamlined, efficient model deployment.

Generally, the RFE is designed using a binary SVM. The RFE uses squared coefficients $w_{j}^{2},\;j=1,2,\ldots ,m$ of weight vector w, where m denotes the total amount of initial features. The factor w is a feature ranking condition, which is expressed in the below Equation (6).

(6) $$w=\sum_{i=1}^{m}\alpha_{i}y_{i}Z_{i}$$

where the input and output pairs are denoted as Z and y, respectively, and α is a positive constant. The coefficient is calculated according to the information gain (contribution percentage of decision for the feature to decide what kind of attack) of the features towards the target label. Hence, the feature with a lesser coefficient is considered as a feature with less information. The RFE executes in an iterative way, whereas the SVM classifier is trained to utilize the remaining features. Subsequently, the ranking condition of features, i.e., $c_{j}=w_{j}^{2}$ is computed, and the features with a lesser ranking condition are discarded using RFE. The aforementioned process is continued until the RFE returns the small feature subset $\left(s\right)$. 

### 3.5. Classification Using LREC

**Figure 2:**
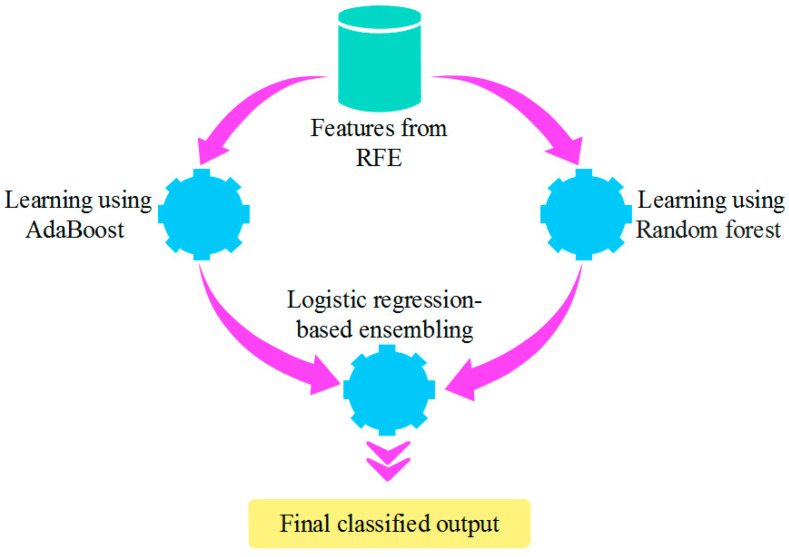
Structure of LREC.

The feature subset from RFE is given as input to the AdaBoost and RF. The two classifiers, AdaBoost and RF, are utilized to compensate for the learning performance of each other in the individual training of subclasses. Here, the AdaBoost concentrates on sequentially enhancing the performance of weak learners, whereas the *RF* develops an ensemble of decision trees for making predictions. Further, the classified outputs from these classifiers are combined using logistic regression to make the final prediction. The logistic regression is utilized as a meta-classifier to weigh and combine the classifications from AdaBoost and RF. The interpretability feature of logistic regression is used for providing insights into the contribution of each base classifier for performing the final classification. Further, logistic regression is used to perform a robust and well-generalized final prediction. Moreover, the logistic regression used in the LREC is generally an ensemble of tree architectures like AdaBoost and RF. Each classifier is trained over the data’s random subset and the feature’s random subset. The generation of multiple trees facilitates minimization of the risk of any single tree overemphasizing specific patterns or falling prey to the base rate fallacy. The structure of the LREC is shown in Figure 2.

#### 3.5.1. Random Forest (RF)

*RF* is generally an enhanced version of bagging where the random selection method is used to construct the trees. Here, the process of training is defined by selecting random attributes. The RFE depends on the following two standards:

(1) A random sampling of training examples while creating a tree;
(2) A random group of features taken while splitting the nodes.

The non-pruning strategy is used to obtain less variance and bias. The idea of integrating multiple trees is used to increase prediction and avoid the over-fitting issue. The X dimension vector is given as input to the *RF*, and Equation (7) expresses the *RF* model $\left(T\right)$ with K amount of decision trees. Each tree in *RF* performs the identification while voting is applied to take decisions. Further, the label identified with a higher number of decision trees is returned as the final prediction.

(7) $$RF=\frac{1}{K}\sum_{i=1}^{K}T\left(s\right)$$

Bootstrap aggregation is used for minimizing the correlation among various decision trees. The robustness and generalization are revamped by generating the decision trees over a dissimilar training subset.

#### 3.5.2. AdaBoost

The AdaBoost algorithm uses the boosting concept, which helps to produce a robust classifier out of the weaker classifiers. AdaBoost can increase the overall effectiveness of ML classifiers by integrating bad classifiers and extracting the prediction value to create a superior classifier known as an ensemble classifier. The AdaBoost classifier minimizes problems related to overfitting and aids in producing better results. It considers the best values of every individual classifier and selects the best values.

#### 3.5.3. Logistic Regression

Logistic regression is an ensemble learning that integrates multiple classifiers such as AdaBoost and *RF* for improving the performance using an iterative ensemble approach. It thus creates an effective classifier. Logistic regression evaluates the relationship between various independent variables and the categorical dependent variable. Further, this logistic regression evaluates the posterior probability $\left(p\right)$ of happening by fitting the data into the logistic function. The primary principle is to fix the weights of classifiers and train the sample in every boosting iteration to precisely identify the class target of the given data. Equation (8) expresses the classification $\left(y^{*}\right)$ of logistic regression.

(8) $$y^{*}=ln\left(\frac{p}{1-p}\right)$$

The steps carried out in the LREC are given in the following Algorithm 1.
**Algorithm 1:** Classification using LRECInput: Features Output: Intrusion Attacks Classification For each feature f in Data X  Perform data normalization  Perform data oversampling   Do Compute X # RFE Feature selection  while   For j=1,2,…N do # N number of features    Update the value of X using Equation (6)  Do (check converged) Initialize training data instance space S For t=1,2,…T do # T is number of base learners  Train a base learners: X → H using the distribution X # Ada boost, RFC and H is trained tree structure   Update the distribution over the training of data End for Compute the final score for the instances of base learners Create the final score based on the meta-learner LR Initialize testing the data instance space S if all the results of d classes S // logistic regression  Return S End if Repeat process Evaluate the performance metrics

## 4. Results and Discussion

The outcomes of the RFE-LREC method are detailed in this section. The RFE-LREC method is developed and implemented using Python 3.7 language, with the system configuration considered during analysis being i5 processor, Windows 10 operating system and 16 GB RAM. The BoT-IoT and TON-IoT datasets are separated in the ratio of 80:20 for training and testing purposes. This 80:20 ratio is considered because it strikes a realistic balance between bias and variance while evaluating the RFE-LREC performance. With an 80% training set, an adequately large sample is obtained to effectively train LREC, while the 20% test set offers an adequate sample to evaluate the LREC’s generalization performance. The RFE-LREC is evaluated using accuracy, recall, precision, F1-score, *TNR* and *MCC*, as expressed in Equations (9)–(14), and also as per FAR and ROC. Additionally, the execution time (ET) and complexity are analyzed for further evaluation.

(9) $$Accuracy=\frac{TP+TN}{TP+FP+TN+FN}$$

(10) $$Recall=\frac{TP}{TP+FN}$$

(11) $$Precision=\frac{TP}{TP+FP}$$

(12) $$F1-score=\frac{2TP}{2TP+FP+FN}$$

(13) $$TNR=\frac{TN}{TN+FP}$$

(14) $$MCC=\frac{TP\times TN-FP\times FN}{\sqrt{\left(TP+FP\right)\left(TP+FN\right)\left(TN+FP\right)\left(TN+FN\right)}}$$

where TP and TN denote true positive and true negative and FP and FN denote false positive and false negative.

### 4.1. Performance Evaluation

The performance of the RFE-LREC is analyzed with different classifiers, oversampling approaches and feature selection approaches. A detailed discussion of the performance evaluation is given in the following sections. 

#### 4.1.1. Performance Evaluation for BoT-IoT Dataset

The different state-of-art classifiers—RF, GNB, Decision Tree (Information Gain), Decision Tree (Gini Index) and GBM—are used for evaluating the LREC. The different classes by the name of DDoS, DoS, Reconnaissance, Normal and Theft are considered for analyzing various classifiers, as shown in Table 1. One of the reasons for such consistent high values of the DoS class is data imbalance. If the dataset is heavily skewed towards the majority class (non-DoS), the model is required to achieve a higher accuracy by just identifying the majority class at many times. Next, the different subcategories—UDP, TCP, Service_Scan, OS_Fingerprint, HTTP, Normal and Keylogging—are additionally considered for evaluating the classifiers, as shown in the Table 2. Zero records in Table 1 and Table 2 convey that there is no test data available for those classes trained due to the imbalance in data, and very few instances are obtainable for such classes. This evaluation shows that the LREC provides better performances than the other classifiers. For example, the LREC obtains 99.99% for DoS class, which is the highest in comparison to the RF, GNB, Decision Tree (Information Gain), Decision Tree (Gini Index) and GBM.

**Figure 3:**
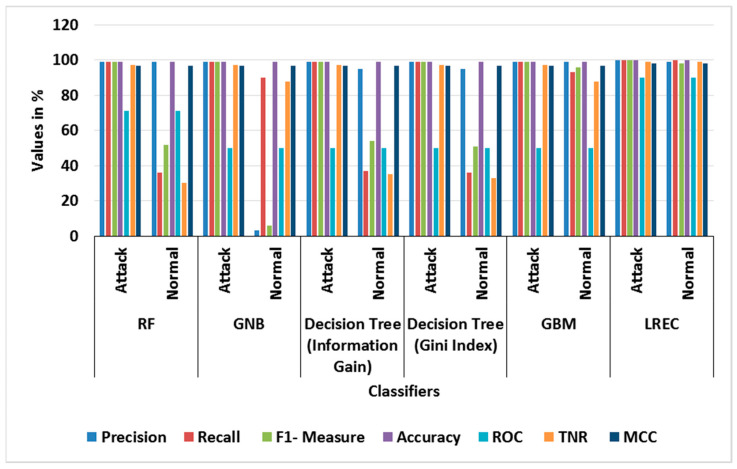
Graph of different classifier performances for binary classes with BoT-IoT.

**Figure 4:**
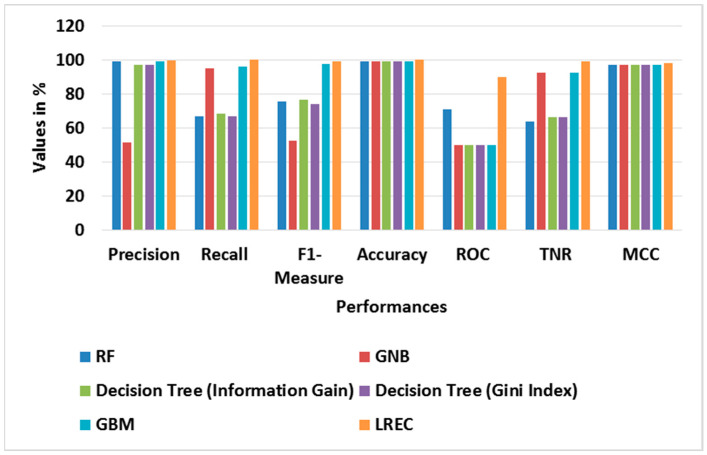
Graph of average performance for different classifiers with BoT-IoT.

Table 3 and Table 4 show the performance evaluation of different classifiers in binary classes, and the attained average results using the BoT-IoT dataset, correspondingly. In the binary class analysis, the classes of Normal and Attack are considered for evaluation. The graph of different classifier performances for binary classes is shown in Figure 3. Likewise, Figure 4 shows the graph of the average performance comparison with different classifiers with the BoT-IoT dataset. This analysis shows that the LREC provides better classification than the other classifiers. For example, the LREC achieves a classification accuracy of 99.99%, which is highest amongst RF, GNB, Decision Tree (Information Gain), Decision Tree (Gini Index) and GBM. The LREC improves classification by combining multiple classifiers, namely *RF* and AdaBoost, through the iterative ensemble approach.

**Figure 5:**
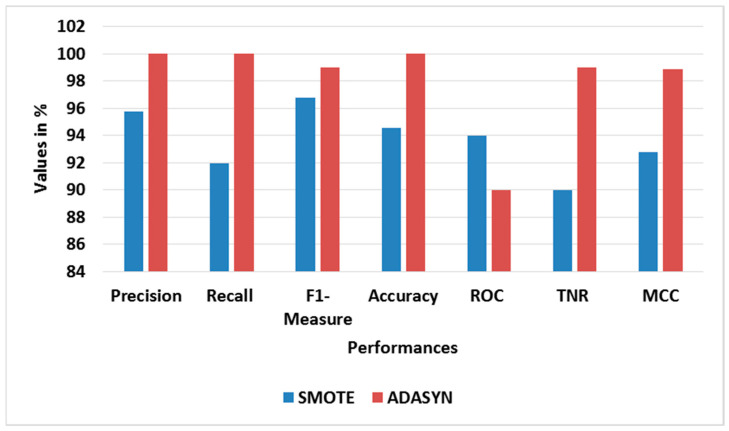
Graph of average performance for different oversampling approaches with BoT-IoT.

**Figure 6:**
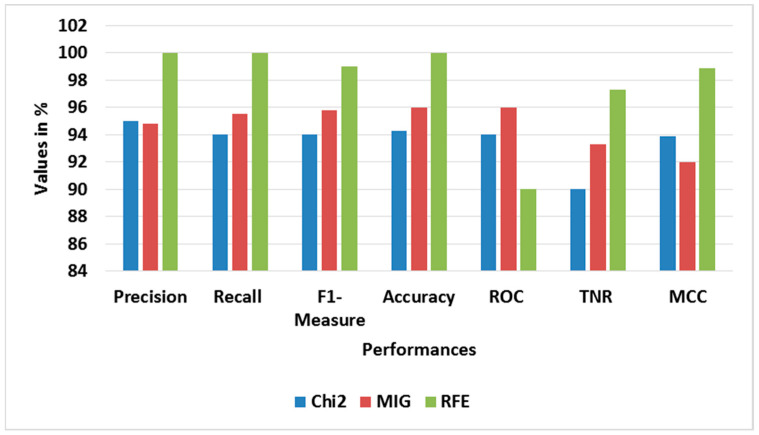
Graph of average performance for feature selection approaches with BoT-IoT.

Table 5 and Table 6 show the performance analysis of different oversampling and feature selection approaches for BoT-IoT, correspondingly. Figure 5 and Figure 6 show the average performance graphs for different oversampling and feature selection approaches, correspondingly. The ADASYN used with RFE-LREC is analyzed with SMOTE, while the RFE is analyzed with Chi-square- and mutual information gain (MIG)-based feature selection approaches. This analysis shows that the ADASYN and RFE exhibit better outputs than the other approaches. The adaptive generation of synthesized samples using ADASYN results in outperformance of the SMOTE. The selection of appropriate features using sequential backward elimination of RFE helps to improve the classification. Moreover, the complexity of $O\left(N\right)$ is common for all classifiers, since it is used in feature selection.

#### 4.1.2. Performance Evaluation for TON-IoT Dataset

**Figure 7:**
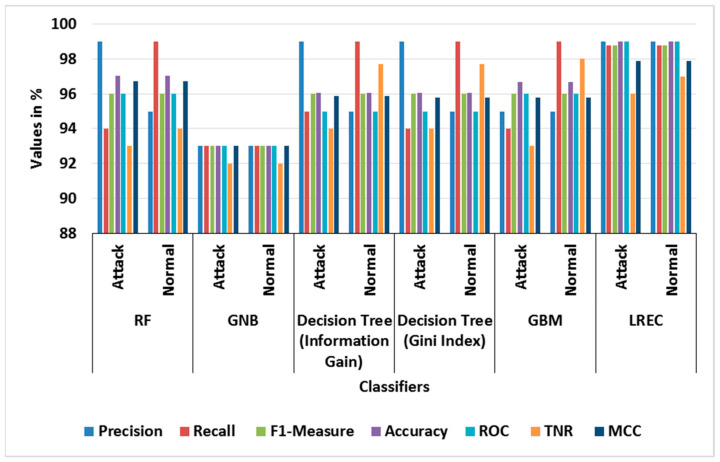
Graph of different classifier performances for binary classes with TON-IoT.

**Figure 8:**
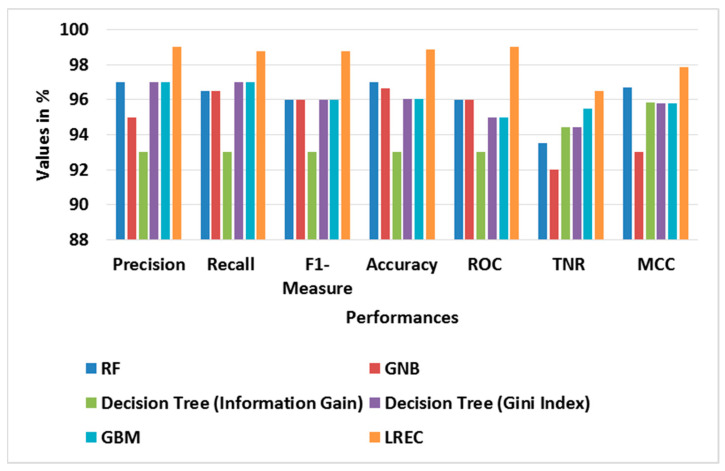
Graph of average performance for different classifiers with TON-IoT.

The evaluation of RFE-LREC using TON-IoT is similar to the previous section. The RFE-LREC for binary classes and average results are shown in the Table 7 and Table 8, respectively. The graph of different classifier results for binary classes is shown in Figure 7. In addition, Figure 8 depicts the graph for the comparison of average performance with different classifiers. The LREC achieves a superior identification of intrusion when contrasted against the RF, GNB, Decision Tree (Information Gain), Decision Tree (Gini Index) and GBM. For instance, the accuracy of LREC for TON-IoT is 97.06%, which is greater when differentiated with the other classifiers.

**Figure 9:**
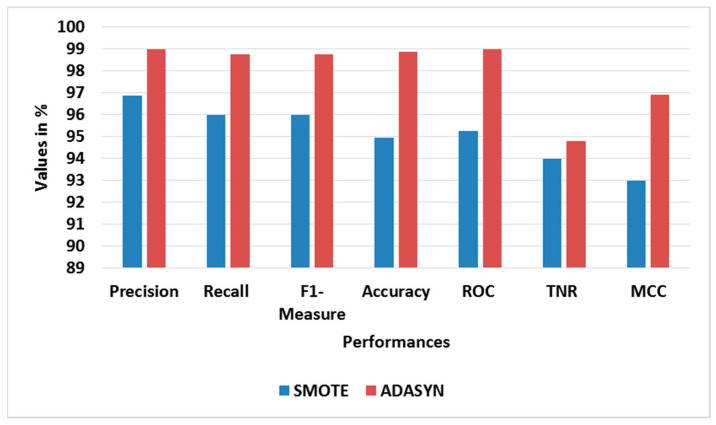
Graph of average performance for different oversampling approaches with TON-IoT.

**Figure 10:**
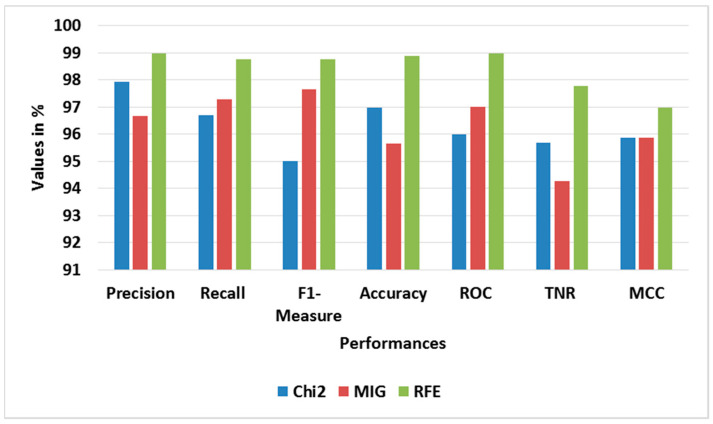
Graph of average performance for feature selection approaches with TON-IoT.

Further, the different oversampling and feature selection approaches are assessed alongside ADASYN with TON-IoT, as shown in Table 9 and Table 10, respectively. An accuracy graph for different oversampling and feature selection approaches with TON-IoT are shown in Figure 9 and Figure 10, respectively. This analysis shows that the ADASYN and RFE display a better performance when differentiated with the remaining approaches. 

### 4.2. Comparative Analysis

**Figure 11:**
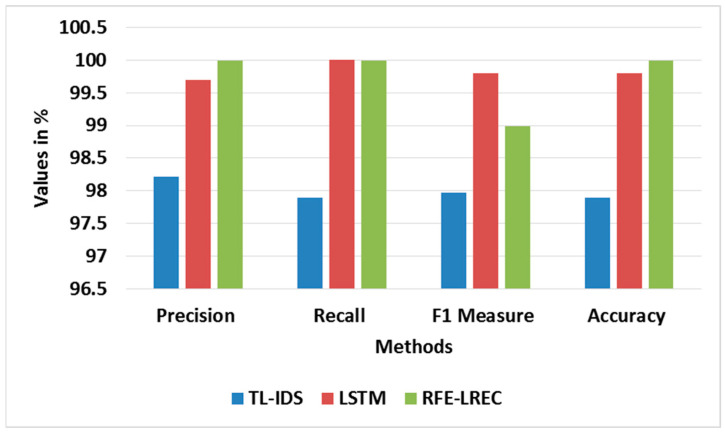
Graph for comparative analysis of BoT-IoT dataset. Adapted from ref. [18,21].

The existing researches, NetFlow-based feature set [16], TL-IDS [18] and LSTM [21] are used to relatively assess the proposed method. The reason for taking these existing researches is that they also contribute towards the classifier. Table 11 shows the comparative analysis of the RFE-LREC with TL-IDS [18] and LSTM [21] in the BoT-IoT, whereas Table 12 shows the comparison of RFE-LREC with NetFlow [16] in TON-IoT. Additionally, the graph of comparative analysis for the BoT-IoT dataset is shown in Figure 11. The LREC accomplishes a better classification by integrating the *RF* and AdaBoost via the iterative ensemble approach. Further, the adaptive generation of synthesized samples using ADASYN is exploited to amplify the classification accuracy. An elimination of the inappropriate features is carried out to amplify the classification.

## 5. Conclusions

The open nature and self-configuring architecture of the IoT causes it to be vulnerable to intrusive cyberattacks. In this research, the LREC, a combination of AdaBoost and RF, is developed for performing an effective classification of intrusion. The ADASYN avoids the issue of data imbalance by adaptively generating the synthesized samples, which is the process of generating a huge amount of samples when there is less density and generating a less number of samples when there is huge density. An inappropriate feature that exists in the overall feature set is eliminated by selecting the features through sequential backward elimination. The interpretability feature of LREC is used for improving the classification performances by combining the AdaBoost and *RF* using an iterative ensemble approach. In addition, logistic regression is deployed to perform robust and generalized final predictions. From the analysis, it is evident that the RFE-LREC exhibits superior outputs than the existing approaches, which are NetFlow, TL-IDS and LSTM. The classification accuracy of RFE-LREC for the BoT-IoT dataset is 99.99%, which is higher when compared to the TL-IDS and LSTM. The developed IDS will be improved by using the regularization approaches to control overfitting in individual trees that further help to enhance the overall performance of the ensemble model for a better prediction.

The proposed algorithm will be enhanced by applying regularization techniques to control overfitting in individual trees, which can improve the overall performance of the ensemble model for better intrusion detection.

## Figures and Tables

**Table 1:** Evaluation of classifiers for BoT-IoT dataset with different classes.

Classifiers	Class	Precision (%)	Recall (%)	F1-Measure (%)	Accuracy (%)	ROC (%)	FAR (%)	TNR (%)	MCC (%)
RF	DDoS	98	96	97	97	98.99	6.87	95	97
	DoS	96	98	97	97	98.99	6.87	97	97
	Reconnaissance	98.99	24	39	97	90	6.87	55	95
	Normal	98.99	97	98	97	90	6.87	97	95
	Theft	98.99	29	44	97	98.99	6.87	45	95
GNB	DDoS	66	96	78	71	98.99	6.15	97	78
	DoS	89	44	59	71	98.99	6.15	79	78
	Reconnaissance	03	90	06	71	50	6.15	95	78
	Normal	89	21	33	71	98.99	6.15	44	78
	Theft	98.99	57	73	71	98.99	6.15	67	78
Decision Tree (Information Gain)	DDoS	97	88	92	91	98.99	5.44	90	92
	DoS	86	96	91	91	50	5.44	99	92
	Reconnaissance	94	31	46	91	90	5.44	55	92
	Normal	94	57	71	91	92	5.44	76	92
	Theft	00	00	00	91	99.99	5.44	01	92
Decision Tree (Gini Index)	DDoS	97	88	92	91	99.99	5.48	89	92
	DoS	86	97	91	91	99.99	5.48	98	92
	Reconnaissance	91	30	45	91	99.99	5.48	45	92
	Normal	95	59	73	91	90	5.48	69	92
	Theft	00	00	00	91	99.99	5.48	02	92
GBM	DDoS	98.99	98.99	98.99	98.99	99.99	7.6	98	98
	DoS	98.99	98.99	98.99	98.99	99.99	7.6	98	98
	Reconnaissance	97	93	94	98.99	50	7.6	98	97.99
	Normal	98.99	98.99	98.99	98.99	90	7.6	98	97.99
	Theft	98.99	98.99	98.99	98.99	99.99	7.6	98	98.76
LREC	DDoS	98.99	99.99	98.99	98.99	99.99	2.0	99	98.99
	DoS	99.99	99.99	99.99	99.99	99.99	2.0	99	98.99
	Reconnaissance	99	99.99	98	99.99	98	2.0	99	98.99
	Normal	99.99	99.99	99.99	99.99	98	2.0	99	98.99
	Theft	99.99	99.99	98	99.99	99.99	2.0	99	98.99

**Table 2:** Evaluation of classifiers for BoT-IoT dataset with different subcategories.

Classifiers	Subcategory	Precision (%)	Recall (%)	F1-Measure (%)	Accuracy (%)	ROC (%)	FAR (%)	TNR (%)	MCC (%)
RF	UDP	98	19	32	98	50	4.2	22	96
	TCP	00	00	00	98	50	4.2	03	96
	Service_Scan	99.99	02	04	98	50	4.2	03	96
	OS_Fingerprint	00	00	00	98	98.99	4.2	02	96
	HTTP	80	97	97	98	98.99	4.2	95	96
	Normal	98.99	98.99	98.99	98	50	4.2	97.98	96
	Keylogging	98.99	98.99	98.99	98	53	4.2	97.98	96
GNB	UDP	17	67	28	98	50	4.28	77	96
	TCP	98.99	50	67	98	98.99	4.28	65	96
	Service_Scan	03	90	06	98	50	4.28	95	96
	OS_Fingerprint	00	00	00	98	50	4.28	03	96
	HTTP	83	20	33	98	98.99	4.28	33	96
	Normal	96	99	98	98	50	4.28	97	96
	Keylogging	98.99	98.99	98.99	98	53	4.28	97	96
Decision Tree (Information Gain)	UDP	88	76	82	99	50	7.6	78	98
	TCP	98.99	36	53	99	50	7.6	44	98
	Service_Scan	95	34	50	99	50	7.6	44	98
	OS_Fingerprint	81	26	39	99	50	7.6	45	98
	HTTP	88	59	71	99	50	7.6	79	98
	Normal	98	98.99	99	99	50	7.6	96.99	98
	Keylogging	98.99	98.99	98.99	99	50	7.6	96.99	98
Decision Tree (Gini Index)	UDP	86	83	84	99	50	5.7	87	97
	TCP	00	00	00	99	50	5.7	02	97
	Service_Scan	83	05	09	99	50	5.7	03	97
	OS_Fingerprint	92	24	38	99	98.99	5.7	32	97
	HTTP	88	62	73	99	98.99	5.7	56	97
	Normal	98	100	99	99	98.99	5.7	98	97
	Keylogging	98.99	98.99	98.99	99	98.99	5.7	98	97
GBM	UDP	98.99	98	99	98.99	50	4.8	96	98
	TCP	98.99	98	99.99	98.99	50	4.8	96	98
	Service_Scan	99	93	96	98.99	96	4.8	92	98
	OS_Fingerprint	93	93	93	98.99	98.99	4.8	92	98
	HTTP	98	98	98	98.99	98.99	4.8	97	98
	Normal	98.99	98.99	98.99	98.99	50	4.8	97	98
	Keylogging	98.99	98.99	98.99	98.99	53	4.8	99	98
LREC	UDP	99.99	99	99.99	99.99	50	0.01	99	99
	TCP	95	99.99	90	99.99	50	0.01	99	99
	Service_Scan	97	99.99	98	99.99	50	0.01	99	99
	OS_Fingerprint	95	94	94	99.99	98.99	0.01	99	99
	HTTP	99	99	99	99.99	98.99	0.01	99	99
	Normal	99.99	99.99	99.99	99.99	98.99	0.01	99	99
	Keylogging	99.99	99.99	99.99	99.99	98.99	0.01	99	99

**Table 3:** Evaluation of classifiers for BoT-IoT dataset with binary classes.

Classifiers	Class	Precision (%)	Recall (%)	F1-Measure (%)	Accuracy (%)	ROC (%)	FAR (%)	TNR (%)	MCC (%)
RF	Attack	98.99	98.99	98.99	98.99	71	7.0	97.07	97
	Normal	98.99	36	52	98.99	71	7.0	30	97
GNB	Attack	98.99	98.99	98.99	98.99	50	5.0	97.07	97
	Normal	03	90	06	98.99	50	5.0	88	97
Decision Tree (Information Gain)	Attack	98.99	98.99	98.99	98.99	50	4.9	97.07	97
	Normal	95	37	54	98.99	50	4.9	35	97
Decision Tree (Gini Index)	Attack	98.99	98.99	98.99	98.99	50	5.7	97.07	97
	Normal	95	36	51	98.99	50	5.7	33	97
GBM	Attack	98.99	98.99	98.99	98.99	50	5.7	97.07	97
	Normal	99	93	96	98.99	50	4.9	88	97
LREC	Attack	99.99	99.99	99.99	99.99	90	4.8	99	98
	Normal	99	99.99	98	99.99	90	4.8	99	98

**Table 4:** Average classification performances for different classifiers with BoT-IoT.

Classifiers	Precision (%)	Recall (%)	F1 Measure (%)	Accuracy (%)	ROC (%)	FAR (%)	TNR (%)	MCC (%)	ET (min)	Complexity
RF	98.99	66.99	75.5	98.99	71.00	7.0	63.53	97	20	O (2M + N)
GNB	51.5	94.99	52.5	98.99	50.00	5.0	92.53	97	25	O (M + N)
Decision Tree (Information Gain)	96.99	68.49	76.5	98.99	50.00	4.9	66.03	97	30	O (M + N)
Decision Tree (Gini Index)	97.00	67.00	74.00	98.99	50.00	5.7	66	97	40	O (M + N)
GBM	98.99	95.99	97.48	98.99	50.00	8.15	92.53	97	55	O (M + N)
LREC	99.49	99.99	98.99	99.99	90.00	4.8	99	98	12	O (M + N)

**Table 5:** Evaluation of different oversampling approaches for BoT-IoT.

Oversampling	Precision (%)	Recall (%)	F1 Measure (%)	Accuracy (%)	ROC (%)	FAR (%)	TNR (%)	MCC (%)
SMOTE	95.79	91.98	96.79	94.58	94.00	5.7	89.99	92.78
ADASYN	99.99	99.99	98.99	99.99	90.00	4.8	98.99	98.88

**Table 6:** Evaluation of different feature selection approaches for BoT-IoT.

Feature Selection	Precision (%)	Recall (%)	F1 Measure (%)	Accuracy (%)	ROC (%)	FAR (%)	TNR (%)	MCC (%)
Chi2	95.00	94.00	94.00	94.28	94.00	7.0	90.00	93.88
MIG	94.79	95.53	95.77	96.00	96.00	5.7	93.33	92.00
RFE	99.99	99.99	98.99	99.99	90.00	4.8	97.33	98.88

**Table 7:** Evaluation of classifiers for TON-IoT dataset with binary classes.

Classifiers	Class	Precision (%)	Recall (%)	F1 Measure (%)	Accuracy (%)	ROC (%)	FAR (%)	TNR (%)	MCC (%)
RF	Attack	98.99	94	96	97.02	96	5.5	93	96.72
	Normal	95	98.99	96	97.02	96	5.5	94	96.72
GNB	Attack	93	93	93	93.00	93	6.8	92	93.00
	Normal	93	93	93	93.00	93	6.8	92	93.00
Decision Tree (Information Gain)	Attack	98.99	95	96	96.06	95	5.5	94	95.86
	Normal	95	98.99	96	96.06	95	5.5	97.69	95.86
Decision Tree (Gini Index)	Attack	98.99	94	96	96.06	95	5.6	94	95.77
	Normal	95	98.99	96	96.06	95	5.6	97.69	95.77
GBM	Attack	95	94	96	96.66	96	5.6	93	95.77
	Normal	95	99	96	96.66	96	5.5	98	95.77
LREC	Attack	98.99	98.77	98.77	98.99	98.99	5.2	96	97.88
	Normal	99	98.77	98.77	98.99	98.99	5.2	97	97.88

**Table 8:** Average classification performances for different classifiers with TON-IoT.

Classifiers	Precision (%)	Recall (%)	F1 Measure (%)	Accuracy (%)	ROC (%)	FAR (%)	TNR (%)	MCC (%)	ET (min)	Complexity
RF	96.99	96.49	96	97.02	96	5.5	93.5	96.72	20	O (2M + N)
GNB	95	96.5	96	96.66	96	5.55	92	93.00	25	O (M + N)
Decision Tree (Information Gain)	93	93	93	93	93	6.8	94.45	95.86	30	O (M + N)
Decision Tree (Gini Index)	96.99	96.99	96	96.06	95	5.5	94.45	95.77	40	O (M + N)
GBM	96.99	96.99	96	96.06	95	5.6	95.5	95.77	55	O (M + N)
LREC	98.99	98.77	98.77	98.88	98.99	5.2	96.5	97.88	12	O (M + N)

**Table 9:** Evaluation of different oversampling approaches for TON-IoT.

Oversampling	Precision (%)	Recall (%)	F1 Measure (%)	Accuracy (%)	ROC (%)	FAR (%)	TNR (%)	MCC (%)
SMOTE	96.87	96.00	95.97	94.96	95.26	5.8	94.00	92.99
ADASYN	98.99	98.77	98.77	98.88	98.99	5.2	94.79	96.89

**Table 10:** Evaluation of different feature selection approaches for TON-IoT.

Feature Selection	Precision (%)	Recall (%)	F1 Measure (%)	Accuracy (%)	ROC (%)	FAR (%)	TNR (%)	MCC (%)
Chi2	97.95	96.70	95.00	96.99	96.00	5.4	95.70	95.88
Mutual information gain	96.66	97.28	97.65	95.66	97.00	5.5	94.28	95.88
RFE	98.99	98.77	98.77	98.88	98.99	5.2	97.77	96.98

**Table 11:** Comparative analysis for BoT-IoT dataset.

Methods	Precision (%)	Recall (%)	F1 Measure (%)	Accuracy (%)
TL-IDS [18]	98.22	97.89	97.97	97.89
LSTM [21]	99.7	100	99.8	99.8
RFE-LREC	99.99	99.99	98.99	99.99

**Table 12:** Comparative analysis for TON-IoT dataset.

Methods	F1 Measure (%)	Accuracy (%)
NetFlow [16]	99	97.86
RFE-LREC	98.77	98.88

## Data Availability

The datasets generated during and/or analyzed during the current study are available in the BoT-IoT (https://staff.itee.uq.edu.au/marius/NIDS_datasets/#RA3, accessed on 3 August 2023) and ToN-IoT (https://staff.itee.uq.edu.au/marius/NIDS_datasets/#RA2, accessed on 3 August 2023) repositories.
